# Impact of mask-wearing on child and adolescent psychosocial development: a systematic review

**DOI:** 10.1093/eurpub/ckac131.043

**Published:** 2022-10-25

**Authors:** A Freiberg, K Horvath, TM Hahne, S Drössler, D Kämpf, A Spura, B Buhs, A Seidler

**Affiliations:** 1 IPAS, TU Dresden, Dresden, Germany; 2 IQTIG, Berlin, Germany; 3 Federal Centre for Health Education, Cologne, Germany

## Abstract

**Background:**

Wearing face masks in public is recommended under certain circumstances in order to prevent infectious diseases transmitted through droplets. The objective was to compile all German and English research results from peer-reviewed journal articles using a sensitive literature search on the effects of mask-wearing for preventing infectious diseases on the psychosocial development of children and adolescents.

**Methods:**

A systematic review was conducted considering different study designs (search period up until 12 July 2021). The risk of bias in the studies was determined using a risk of bias procedure. A descriptive-narrative synthesis of the results was performed. A search update will be conducted shortly before the conference.

**Results:**

Thirteen studies were included, and the overall risk of bias was estimated to be high in all primary studies. There are some indications from the included surveys that children, adolescents, and their teachers in (pre)schools perceived facial expression processing as impaired due to mask-wearing, which were confirmed by several experimental studies. Two studies reported psychological symptoms like anxiety and stress as well as concentration and learning problems due to wearing a mask during the COVID-19 pandemic. One survey study during the 2002/2003 SARS pandemic examined oral examination performance in English as a foreign language and showed no difference between the “mask” and “no mask” conditions. The results are preliminary and may be extended due to the search update.

**Conclusions:**

Only little evidence can be derived on the effects of face masks on different developmental areas of children and adolescents based on the small number of studies. There is a lack of research data regarding the following outcomes: psychological development, language development, emotional development, social behaviour, school success, and participation. Further cluster-randomized controlled trials or longitudinal studies are required.

**Key messages:**

• Empirical and experimental evidence shows that mask-wearing impairs facial expression among children, adolescents, and teachers in (pre)schools.

• Research on the following developmental areas is missing: psychological development, language development, emotional development, social behaviour, school success, and participation.

